# Supplementary material for: Infection and telomere length: a systematic review protocol
Source: BMJ Open. 2024 Apr 23;14(4):e081881. doi: 10.1136/bmjopen-2023-081881 (PMC11043687; doi:10.1136/bmjopen-2023-081881)
Supplement: Supplementary data [file bmjopen-2023-081881supp001.pdf]

Appendix

Medline search strategy (no limits):

1 (infect* or pathogen or virus* or viral or bacteri* or parasit* or communicable disease*).mp.

2 exp Infections/

3 (telomer* or TTAGGG* or chromosome end* or chromosome cap* or end-replication problem or end-replication malfunction* or end-replication issue* or end-replication impairment* or end-replication failure*).ti,ab.

4 Telomere Shortening/

5 Telomere/

6 ((case* adj5 control*) or (case adj3 comparison*) or control group* or cohort or longitudinal or prospective or retrospective).ti,ab. or "clinical trial".pt. or "clinical trial, phase i".pt. or "clinical trial, phase ii".pt. or clinical trial, phase iii.pt. or clinical trial, phase iv.pt. or controlled clinical trial.pt. or "multicenter study".pt. or "randomi?ed controlled trial".pt. or ((randomi?ed adj7 trial*) or (controlled adj3 trial*) or (clinical adj2 trial*) or ((single or doubl* or tripl* or treb*) and (blind* or mask*))).ti,ab,kw. or ("4 arm" or "four arm").ti,ab,kw. or (cross-sectional or prevalence or transversal).ti,ab,kw. or mendelian randomi?ation.ti,ab. or control patients.mp. or control subjects.mp. or control participants.mp. or patient*.ti,ab. or subjects.ti,ab. or Case-Control Studies/ or Control Groups/ or Matched-Pair Analysis/ or Cohort Studies/ or Longitudinal Studies/ or Follow-Up Studies/ or Prospective Studies/ or Retrospective Studies/ or Double-Blind Method/ or Clinical Trials as Topic/ or Clinical Trials, Phase I as Topic/ or Clinical Trials, Phase II as Topic/ or Clinical Trials, Phase III as Topic/ or Clinical Trials, Phase IV as Topic/ or Controlled Clinical Trials as Topic/ or Randomized Controlled Trials as Topic/ or "Early Termination of Clinical Trials"/ or Multicenter Studies as Topic/ or Cross-Sectional Studies/ or Prevalence/ or Epidemiologic Studies/ or Mendelian Randomization Analysis/ or Observational Study/

7 1 or 2

8 3 or 4 or 5

9 6 and 7 and 8
