# Supplementary material for: Infection and telomere length: a systematic review protocol
Source: BMJ Open. 2024 Apr 23;14(4):e081881. doi: 10.1136/bmjopen-2023-081881 (PMC11043687; doi:10.1136/bmjopen-2023-081881)
Supplement: Author's manuscript [file bmjopen-2023-081881.draft_revisions.pdf]

BMJ Open

BMJ Open is committed to open peer review. As part of this commitment we make the peer review history of every article we publish publicly available.

When an article is published we post the peer reviewers' comments and the authors' responses online. We also post the versions of the paper that were used during peer review. These are the versions that the peer review comments apply to.

The versions of the paper that follow are the versions that were submitted during the peer review process. They are not the versions of record or the final published versions. They should not be cited or distributed as the published version of this manuscript.

BMJ Open is an open access journal and the full, final, typeset and author-corrected version of record of the manuscript is available on our site with no access controls, subscription charges or pay-per-view fees (http://bmjopen.bmj.com).

If you have any questions on BMJ Open's open peer review process please email
info.bmjopen@bmj.com

BMJ Open

Infection and telomere length: a systematic review protocol

Journal:	*BMJ Open*
Manuscript ID	bmjopen-2023-081881
Article Type:	Protocol
Date Submitted by the Author:	17-Nov-2023

Complete List of Authors:	Tunnicliffe, Louis; London School of Hygiene & Tropical Medicine, Faculty of Epidemiology & Population Health<br>Muzambi, Rutendo; London School of Hygiene and Tropical Medicine, Faculty of Epidemiology & Population Health<br>Bartlett, Jonathan; London School of Hygiene & Tropical Medicine, Faculty of Epidemiology & Population Health<br>Howe, Laura; University of Bristol, Social Medicine<br>Abdul Basit, Khalid; London School of Hygiene and Tropical Medicine, Faculty of Epidemiology & Population Health<br>Warren-Gash, Charlotte; London School of Hygiene and Tropical Medicine, Non-communicable disease epidemiology

Keywords:	Aging, INFECTIOUS DISEASES, NEUROLOGY

SCHOLARONE™
Manuscripts

Infection and telomere length: a systematic review protocol

Authors: Louis Tunnicliffe[1], Rutendo Muzambi[1], Jonathan W. Bartlett[1], Laura D. Howe[2], Khalid Abdul Basit[1], Charlotte Warren-Gash[1]

1. Faculty of Epidemiology & Population Health, London School of Hygiene & Tropical Medicine, Keppel Street, London, UK

2. MRC Integrative Epidemiology Unit, Department of Population Health Sciences, Bristol Medical School, University of Bristol, Oakfield House, Oakfield Grove, Bristol, UK

Corresponding author:
Louis Tunnicliffe Room 256, LSHTM, Keppel Street, London WC1E 7HT Email: louis.tunnicliffe@lshtm.ac.uk Tel: +44(0) 20 7636 8636

Email addresses of protocol authors (in same order as author list above): louis.tunnicliffe@lshtm.ac.uk, rutendo.muzambi@lshtm.ac.uk, jonathan.bartlett1@lshtm.ac.uk, laura.howe@bristol.ac.uk, khalid.abdul-basit@lshtm.ac.uk, charlotte.warren-gash1@lshtm.ac.uk

Abstract: 242/300

Word count: 2102/ 4000

References: 28

For peer review only

Abstract

Introduction- Telomeres are a measure of cellular aging with potential links to diseases such as cardiovascular diseases and cancer. Studies have shown that some infections may be associated with telomere shortening, but whether an association exists across all types and severities of infections and in which populations is unclear. Therefore we aim to collate available evidence to enable comparison and to inform future research in this field.

Methods and analysis- We will search for studies involving telomere length and infection in various databases including MEDLINE (Ovid interface), EMBASE (Ovid interface), Web of Science, Scopus, Global Health, and the Cochrane Library. For grey literature the British Library of electronic theses databases (ETHOS) will be explored. We will not limit by study type, geographic location, infection type or method of outcome measurement. Two researchers will independently carry out study selection, data extraction, and risk of bias assessment using the ROB2 and ROBINS-E tools. Overall quality of the studies will be determined using the Grading of Recommendations, Assessment, Development, and Evaluations criteria. We will also evaluate study heterogeneity with respect to study design, exposure and outcome measurement and if there is sufficient homogeneity, a meta-analysis will be conducted. Otherwise we will provide a narrative synthesis with results grouped by exposure category and study design.

Ethics and dissemination- The present study does not require ethical approval. Results will be disseminated via publishing in a peer-reviewed journal and conference presentations.

PROSPERO registration number CRD42023444854

Strengths and limitations of this study

- This study will be conducted in adherence to the established Preferred Reporting Items for Systematic Reviews and Meta-analyses Protocols (PRISMA-P) statement
- Study selection, data extraction, risk of bias assessment and certainty assessment will be performed by two independent reviewers using well established guidelines and methods
- We will search a range of relevant databases including published and grey literature
- One limitation is that suspected heterogeneity with respect to exposure type and outcome measurement may mean a meta-analysis is not possible

Introduction

Rationale

Telomeres are structures found at the ends of chromosomes which are composed of repetitive DNA sequences and protective proteins. Their primary role is to shield the genomic DNA from being recognized as damaged or broken to prevent processes such as DNA end-joining, DNA recombination, or DNA repair that could lead to chromosome instability (1).

The DNA replication machinery in cells cannot fully copy the DNA at the extreme ends of linear chromosomes, which results in the gradual shortening of chromosome ends with each cell division (1). Eukaryotic cells address this via an enzyme called telomerase which acts to replenish the chromosome ends (2). However in many human cell types, the levels of telomerase (or its activity on telomeres) are limited. This combined with factors such as nuclease action, chemical damage, and DNA replication stress results in the continuous shortening of telomeres throughout a person's lifespan. For this reason, telomere length is used as a measure of biological aging (1).

When telomeres reach a critical length or experience significant damage, a prolonged DNA damage response is triggered. This results in changes to gene expression patterns and leads to cellular senescence (3). The specific outcomes of senescence are thought to vary depending on cell type (1). It has been extensively documented that inflammation plays a significant role in the progression of diseases like cardiovascular disease, chronic kidney disease and Alzheimer's disease (4). Given that immune cell senescence induces pro-inflammatory processes, telomere attrition in immune cells becomes relevant to the development of these conditions (1). Furthermore there is evidence to suggest that telomere shortening is associated with increased incidence of various diseases including Alzheimer's disease (5) and cardiovascular disease (6) even after adjusting for age. The idea that shorter telomeres are a potential risk factor for age associated diseases is reinforced by the fact that inherited telomere syndromes, where

individuals are genetically pre-disposed to have short telomeres, are characterised by phenotypes of accelerated aging, including a host of age-associated diseases (7).

Telomere length has been shown to be associated with lifestyle and environmental factors (1). For example, early-life connections between stress and telomeres are evident (8). Infections are another factor which could influence telomere length via pathways such as inflammation and oxidative stress (9)(10). However, there is lack of robust evidence relating to the association between infection and telomere length.

While some infections have been studied in relation to telomere length, existing studies differ in the types and severities of infections studied, definitions used and use differing measures of telomere length, making pooling evidence across studies challenging (9,10)(12-16). Moreover, cross-sectional studies are the most abundant study type in this field; meaning there is a potential for reverse causality. Despite the heterogeneity, some evidence suggests that associations between some persistent viral infections such as Cytomegalovirus and Herpes simplex virus type-1 were associated with reduced telomere length or telomere attrition (9,10,13,14,16). Current gaps in research include establishing whether infections as a whole are risk factors for reduced telomere length and whether pathogen type, severity, and infection site are associated with telomere length. It is plausible that telomere attrition could act as a mechanism through which infections mediate effects on age-related diseases and thereby represent a target for intervention. However, the degree to which any associations are causal remains unclear A systematic review looking at the potential association between infection and telomere length is needed as no prior reviews have been conducted and they are crucial for identifying research gaps and informing the design of future studies.

Objectives

This systematic review aims to comprehensively summarize all existing literature on the association between infections (by type, site, severity) and telomere length or attrition across a broad range of study designs (see eligibility criteria) in adult humans. We aim to establish whether there is an association between infection and telomere length to inform future studies.

Research questions

1. Is there an association between infections and telomere length or attrition?

2. Is infection type, site, severity associated with telomere length or attrition?

3. Is preventing or treating infections associated with telomere length or attrition?

Methods and analysis

The current protocol for our systematic review adheres to the guidelines provided by the Preferred Reporting Items for Systematic Reviews and Meta-analyses Protocols (PRISMA-P) statement and has been registered in the PROSPERO (registration number CRD42023444854) (17,18). Any modifications to the protocol will be documented and updated on PROSPERO.

We intend to follow the PRISMA statement for reporting the systematic review and if applicable employ the Meta-analysis of Observational Studies in Epidemiology statement for the reporting of any potential meta-analysis (19,20).

Search strategy

We will perform a comprehensive search strategy encompassing both published studies and grey literature. Published studies will be sought from six electronic databases, namely MEDLINE (Ovid interface), EMBASE (Ovid interface), Web of Science, Scopus, Global Health, and the Cochrane Library. For grey literature the British Library of electronic theses databases (ETHOS) will be explored. Additionally, the reference lists of included papers will be manually searched to identify any additional relevant studies.

We constructed a preliminary Medline search using three concepts namely 1. infections 2. telomere length and 3. human study type, these search concepts were combined using the Boolean operator 'AND'. Our search involved combining key words with database-specific subject headings and this search can be found in the appendix. This search was created and translated across databases with support from a librarian at the London School of Hygiene and Tropical Medicine. No restrictions were placed on the geographical location, language or date of publication of the studies. We will aim to translate any potentially relevant non-English language studies.

Selection process

We will utilize reference management software EndNote (version X8.0.2) for storing our search results. We will conduct de-duplication using the automated feature and subsequently inspect the results to identify and eliminate any duplicate entries manually.

For the selection of studies, two researchers will independently assess all titles and abstracts to determine their agreement with the eligibility criteria described below. Reviewers will discuss and agree which articles should go to full text review. We will then obtain the full texts and the process will be repeated. In the event of discrepancies between the reviewers, we will discuss these and if necessary a third reviewer will be consulted. All reasons for excluding studies will be documented at the full text review stage and our study selection process will be illustrated using the PRISMA flow diagram

(19). If multiple papers stem from the same study population then we will include the paper that encompasses the largest sample size and provides the most comprehensive exposure and outcome details.

Eligibility criteria

Studies will be eligible for inclusion in the present study if they meet the criteria below:

Study characteristics

To capture all potentially relevant designs, we will include cross-sectional studies, case control studies, cohort studies, randomised control trials (of vaccination or infection treatment) and Mendelian randomisation studies.

We will include studies of any setting and time-frame.

Population

We will include studies with adults aged ≥18 years from any geographic area and any healthcare / study environment. Animal studies will be excluded.

Exposure

The exposure group will be individuals exposed to infection (i.e. any pathogen, site, severity, type e.g. acute or chronic). Infection diagnosis could be defined through electronic healthcare records (e.g. using ICD-10 or Read coded diagnoses), self-report, antibody measures or other laboratory markers of infection (e.g. PCR). For Mendelian randomisation studies, exposure will be individuals who carry the genetic variants associated with infection and for randomised controlled trials the exposure would be people receiving a vaccine or treatment for infection.

Comparators

The comparator group will vary by study type. For cross-sectional and cohort studies the comparator group will be individuals unexposed to infection. For case-control studies the comparator group is individuals with normal telomere length. For Mendelian randomisation studies, comparators will be individuals who do not carry the genetic variants associated with infection. Finally, for randomised controlled trials the comparator would be people not receiving vaccine or treatment for infection.

Outcome

The outcome will be (i) telomere length (ii) telomere attrition for longitudinal studies.

We will be inclusive in how outcome is measured for example; measured by rate of change, continuous measures or binary measures.

We will include studies with any valid method of ascertainment measurement of telomere length. These include PCR (Polymerase Chain Reaction) methods, TRF (Terminal Restriction Fragment) analysis, a variety of FISH (Fluorescence In Situ Hybridization) methods, STELA (Single TElomere Length Analysis), and TeSLA (Telomere Shortest Length Assay) (21).

Data Collection Process

Two independent researchers will extract information from the selected papers using a piloted data extraction form. The first reviewer will conduct the data extraction in full whereas the second reviewer will extract data on a 10% random sample of the selected studies. In cases where essential data are missing, we will contact authors to request the necessary information.

Data Items

To create our data extraction form, we will adopt the Population, Exposure, Comparator, Outcomes, and Study Characteristics (PECOS) framework (22). Our data extraction will encompass the following elements:

1. Population: This section will include information about the population under study, such as age (mean, median, or range), gender distribution, and the criteria used for inclusion and exclusion e.g. health conditions, location of residence.

2. Exposure: We will extract details regarding the definition of the exposure, the type of infection involved, whether it relates to hospitalized infection, its acute or chronic nature, and the number of individuals exposed.

3. Comparators: Information related to comparators will encompass their identification, definition, and the count of comparators used in the study.

4. Outcomes: We will collect data on the type of measurement used for telomere length (e.g., binary or continuous) and the number of participants who experienced the specified outcome.

5. Study Characteristics: This section will provide essential details about the study, including the authors' names, the study's title, publication year, study design, healthcare setting, country where the study was conducted, sample size, and the duration of follow-up.

Furthermore, we will document any collected covariates and effect modifiers and ensure that both unadjusted and adjusted effect estimates and accompanying 95% confidence intervals are included in our data extraction process. We will also include the results of sub-group analyses e.g. by age and sex.

Assessing Study Bias

Two researchers will independently assess bias following the Cochrane collaboration approach (23) using the ROBINS-E tool (24) for observational studies and the ROB2 (25) tool for randomized controlled trials (RCTs). Both tools will be pilot tested. The ROBINS-E tool will involve evaluating the risk of bias in the following domains: confounding, measurement of the exposure, selection of participants into the study (or into the analysis), post-exposure interventions, missing data, measurement of the outcome, selection of the reported result. The ROB2 tool will involve evaluating bias related to the following domains: the randomisation process, deviations from intended interventions, missing outcome data, measurement of the outcome, selection of the reported result.

Data Synthesis

We will categorize studies based on exposure (infection/pathogen type and site, severity, acute or chronic status), outcome type (length or attrition), study type and summarize data in predefined tables. Our primary analyses will focus on the main exposures of any infection, any vaccination and any antimicrobial treatment. We will then conduct secondary analyses of infection type and severity.

A meta-analysis will be considered feasible if there are at least five homogeneous studies in terms of design, exposure (infection/pathogen type, severity), outcome (telomere length measurement technique) as well as the time between exposure and outcome measurement. Pooled effect measures (odds ratios, risk ratios or hazard ratios and corresponding 95% confidence intervals) of the studies will be computed and study results displayed in Forest plots.

Statistical heterogeneity will be assessed using forest plots, χ^2 test, and I^2 statistic and a random effects meta-analysis will be conducted (26-27). Publication bias and small study effects will be assessed with funnel plots if there are ≥10 eligible studies. If a meta-analysis is unfeasible then a narrative synthesis will be provided with results grouped by exposure.

Certainty assessment

We will use the Grading of Recommendations, Assessment, Development and Evaluation (GRADE) tool to evaluate evidence quality for each outcome (28). Domains considered include risk of bias (determined as described above), inconsistency, indirectness, imprecision, and publication bias. The evidence will be categorized as high, moderate, low, or very low.

Ethics and Dissemination

The present study does not require ethical approval. Results will be submitted for publication in a peer-reviewed journal and may be presented at relevant conferences. The review will highlight research gaps and future directions in this field.

Patient and Public Involvement

Patients and the public were not involved in any way.

References

1. Blackburn EH, Epel ES, Lin J. Human telomere biology: A contributory and interactive factor in aging, disease risks, and protection. Science. 2015 Dec 4;350(6265):1193-8. doi: 10.1126/science.aab3389. PMID: 26785477.

2. Blackburn EH, Greider CW, Szostak JW. Telomeres and telomerase: the path from maize, Tetrahymena and yeast to human cancer and aging. Nat Med. 2006 Oct;12(10):1133-8. doi: 10.1038/nm1006-1133. PMID: 17024208.
3. Abdallah P, Luciano P, Runge KW et al. A two-step model for senescence triggered by a single critically short telomere. Nat Cell Biol. 2009 Aug;11(8):988-93. doi: 10.1038/ncb1911. Epub 2009 Jul 13. Erratum in: Nat Cell Biol. 2010 May;12(5):520. PMID: 19597486; PMCID: PMC4025917.
4. Ferrucci L, Fabbri E. Inflammageing: chronic inflammation in ageing, cardiovascular disease, and frailty. Nat Rev Cardiol. 2018 Sep;15(9):505-522. doi: 10.1038/s41569-018-0064-2. PMID: 30065258; PMCID: PMC6146930.
5. Cai Z, Yan LJ, Ratka A. Telomere shortening and Alzheimer's disease. Neuromolecular Med. 2013 Mar;15(1):25-48. doi: 10.1007/s12017-012-8207-9. Epub 2012 Nov 16. PMID: 23161153.
6. Zhan Y, Hägg S. Telomere length and cardiovascular disease risk. Curr Opin Cardiol. 2019 May;34(3):270-274. doi: 10.1097/HCO.0000000000000613. PMID: 30747731.
7. Walne AJ, Dokal I. Advances in the understanding of dyskeratosis congenita. Br J Haematol. 2009 Apr;145(2):164-72. doi: 10.1111/j.1365-2141.2009.07598.x. Epub 2009 Feb 4. PMID: 19208095; PMCID: PMC2882229.
8. Price LH, Kao HT, Burgers DE et al. Telomeres and early-life stress: an overview. Biol Psychiatry. 2013 Jan 1;73(1):15-23. doi: 10.1016/j.biopsych.2012.06.025. Epub 2012 Jul 24. PMID: 22831981; PMCID: PMC3495091.
9. Dowd JB, Bosch JA, Steptoe A et al. Persistent Herpesvirus Infections and Telomere Attrition Over 3 Years in the Whitehall II Cohort. J Infect Dis. 2017 Sep 1;216(5):565-572. doi: 10.1093/infdis/jix255. PMID: 28931225; PMCID: PMC5853283.
10. Noppert GA, Feinstein L, Dowd JB et al. Pathogen burden and leukocyte telomere length in the United States. Immun Ageing. 2020 Nov 19;17(1):36. doi: 10.1186/s12979-020-00206-9. PMID: 33292353; PMCID: PMC7677839.
11. McKeown RE. The Epidemiologic Transition: Changing Patterns of Mortality and Population Dynamics. Am J Lifestyle Med. 2009 Jul 1;3(1 Suppl):19S-26S. doi: 10.1177/1559827609335350. PMID: 20161566; PMCID: PMC2805833.
12. Huang D, Lin S, He J et al. Association between COVID-19 and telomere length: A bidirectional Mendelian randomization study. J Med Virol. 2022 Nov;94(11):5345-5353. doi: 10.1002/jmv.28008. Epub 2022 Jul 29. PMID: 35854470; PMCID: PMC9349767.
13. Pańczyszyn A, Boniewska-Bernacka E, Głąb G. Telomere length in leukocytes and cervical smears of women with high-risk human papillomavirus (HR HPV) infection. Taiwan J Obstet Gynecol. 2020 Jan;59(1):51-55. doi: 10.1016/j.tjog.2019.11.007. PMID: 32039800.
14. Auld E, Lin J, Chang E et al. HIV Infection Is Associated with Shortened Telomere Length in Ugandans with Suspected Tuberculosis. PLoS One. 2016 Sep 21;11(9):e0163153. doi: 10.1371/journal.pone.0163153. PMID: 27655116; PMCID: PMC5031464.
15. Muhsen K, Sinnreich R, Merom D et al. Helicobacter pylori infection, serum pepsinogens as markers of atrophic gastritis, and leukocyte telomere length: a population-based study. Hum Genomics. 2019 Jul 22;13(1):32. doi: 10.1186/s40246-019-0217-3. PMID: 31331390; PMCID: PMC6647065.

16. van Baarle D, Nanlohy NM, Otto S et al. Progressive telomere shortening of Epstein-Barr virus-specific memory T cells during HIV infection: contributor to exhaustion? J Infect Dis. 2008 Nov 1;198(9):1353-7. doi: 10.1086/592170. PMID: 18816191.
17. Moher D, Shamseer L, Clarke M et al; PRISMA-P Group. Preferred reporting items for systematic review and meta-analysis protocols (PRISMA-P) 2015 statement. Syst Rev. 2015 Jan 1;4(1):1. doi: 10.1186/2046-4053-4-1. PMID: 25554246; PMCID: PMC4320440.
18. Booth A, Clarke M, Dooley G et al. The nuts and bolts of PROSPERO: an international prospective register of systematic reviews. Syst Rev. 2012 Feb 9;1:2. doi: 10.1186/2046-4053-1-2. PMID: 22587842; PMCID: PMC3348673.
19. Moher D, Liberati A, Tetzlaff J et al; PRISMA Group. Preferred reporting items for systematic reviews and meta-analyses: the PRISMA statement. PLoS Med. 2009 Jul 21;6(7):e1000097. doi: 10.1371/journal.pmed.1000097. Epub 2009 Jul 21. PMID: 19621072; PMCID: PMC2707599.
20. Stroup DF, Berlin JA, Morton SC et al. Meta-analysis of observational studies in epidemiology: a proposal for reporting. Meta-analysis Of Observational Studies in Epidemiology (MOOSE) group. JAMA. 2000 Apr 19;283(15):2008-12. doi: 10.1001/jama.283.15.2008. PMID: 10789670.
21. Montpetit AJ, Alhareeri AA, Montpetit M et al. Telomere length: a review of methods for measurement. Nurs Res. 2014 Jul-Aug;63(4):289-99. doi: 10.1097/NNR.0000000000000037. PMID: 24977726; PMCID: PMC4292845.
22. Morgan RL, Whaley P, Thayer KA et al. Identifying the PECO: A framework for formulating good questions to explore the association of environmental and other exposures with health outcomes. Environ Int. 2018 Dec;121(Pt 1):1027-1031. doi: 10.1016/j.envint.2018.07.015. Epub 2018 Aug 27. PMID: 30166065; PMCID: PMC6908441.
23. Higgins JP, Altman DG, Gøtzsche PC et al; Cochrane Bias Methods Group; Cochrane Statistical Methods Group. The Cochrane Collaboration's tool for assessing risk of bias in randomised trials. BMJ. 2011 Oct 18;343:d5928. doi: 10.1136/bmj.d5928. PMID: 22008217; PMCID: PMC3196245.
24. Higgins J, Morgan R, Rooney A et al; ROBINS-E Development Group. Risk Of Bias In Non-randomized Studies - of Exposure (ROBINS-E). Launch version, 20 June 2023. Available from: https://www.riskofbias.info/welcome/robins-e-tool.
25. Sterne JAC, Savović J, Page MJ et al. RoB 2: a revised tool for assessing risk of bias in randomised trials. BMJ 2019; 366: l4898.
26. Riley RD, Higgins JP, Deeks JJ. Interpretation of random effects meta-analyses. BMJ. 2011 Feb 10;342:d549. doi: 10.1136/bmj.d549. PMID: 21310794.
27. Higgins J , Green S . Section 9.5.2. identifying and measuring heterogeneity in Cochrane Handbook for systematic reviews of interventions, 2011. Available: https://handbook-5-1.cochrane.org/chapter_9/9_5_2_identifying_and_measuring_heterogeneity.htm [accessed 30 Oct 2023]
28. Guyatt GH, Oxman AD, Vist GE et al; GRADE Working Group. GRADE: an emerging consensus on rating quality of evidence and strength of recommendations. BMJ. 2008 Apr 26;336(7650):924-6. doi: 10.1136/bmj.39489.470347.AD. PMID: 18436948; PMCID: PMC2335261.

Authors contributions:

Louis Tunnicliffe wrote the present paper and constructed the search strategy with the help of a London School of Hygiene & Tropical Medicine librarian. The writing and search strategy was reviewed by Professor Charlotte Warren-Gash, Doctor Rutendo Muzambi, Professor Laura Howe and Professor Jonathan Bartlett. Louis will be conducting the search, data extraction as well as risk of bias and certainty assessments alongside another independent reviewer namely Doctor Khalid Abdul Basit.

Funding statement:

This work was supported by the Wellcome Trust. Grant number: Wellcome Career Development Award 225868/Z/22/Z to Charlotte Warren-Gash.

Competing interests statement:

There are no competing interests to declare.

Appendix

Medline search strategy (no limits):

1 (infect* or pathogen or virus* or viral or bacteri* or parasit* or communicable disease*).mp.

2 exp Infections/

3 (telomer* or TTAGGG* or chromosome end* or chromosome cap* or end-replication problem or end-replication malfunction* or end-replication issue* or end-replication impairment* or end-replication failure*).ti,ab.

4 Telomere Shortening/

5 Telomere/

6 ((case* adj5 control*) or (case adj3 comparison*) or control group* or cohort or longitudinal or prospective or retrospective).ti,ab. or "clinical trial".pt. or "clinical trial, phase i".pt. or "clinical trial, phase ii".pt. or clinical trial, phase iii.pt. or clinical trial, phase iv.pt. or controlled clinical trial.pt. or "multicenter study".pt. or "randomi?ed controlled trial".pt. or ((randomi?ed adj7 trial*) or (controlled adj3 trial*) or (clinical adj2 trial*) or ((single or doubl* or tripl* or treb*) and (blind* or mask*))).ti,ab,kw. or ("4 arm" or "four arm").ti,ab,kw. or (cross-sectional or prevalence or transversal).ti,ab,kw. or mendelian randomi?ation.ti,ab. or control patients.mp. or control subjects.mp. or control participants.mp. or patient*.ti,ab. or subjects.ti,ab. or Case-Control Studies/ or Control Groups/ or Matched-Pair Analysis/ or Cohort Studies/ or Longitudinal Studies/ or Follow-Up Studies/ or Prospective Studies/ or Retrospective Studies/ or Double-Blind Method/ or Clinical Trials as Topic/ or Clinical Trials, Phase I as Topic/ or Clinical Trials, Phase II as Topic/ or Clinical Trials, Phase III as Topic/ or Clinical Trials, Phase IV as Topic/ or Controlled Clinical Trials as Topic/ or Randomized Controlled Trials as Topic/ or "Early Termination of Clinical Trials"/ or Multicenter Studies as Topic/ or Cross-Sectional Studies/ or Prevalence/ or Epidemiologic Studies/ or Mendelian Randomization Analysis/ or Observational Study/

7 1 or 2

8 3 or 4 or 5

9 6 and 7 and 8

PRISMA-P checklist

Section 1: Administrative information

Title

Item 1a: Identification. Identify the report as a protocol of a systematic review:
Title

Item 1b: Update. If the protocol is for an update of a previous systematic review, identify as such
N/A

Registration

Item 2. If registered, provide the name of the registry (such as PROSPERO) and registration number
See below abstract, PROSPERO registration number CRD42023444854

Authors

Item 3a: Contact information. Provide name, institutional affiliation, and email address of all protocol authors; provide physical mailing address of corresponding author
Title page

Item 3b: Contributions. Describe contributions of protocol authors and identify the guarantor of the review
Author contributions section

Amendments
Item 4 If the report represents an amendment of a previously completed or published protocol, identify as such and indicate what changes were made; otherwise state plan for documenting important protocol amendments
N/A

Support
Item 5a: Sources. Indicate sources of financial or other support for the review
Funding statement section

Item 5b: Sponsor. Provide name of the review funder and/or sponsor
Funding statement section

Item 5c: Role of sponsor and/or funder. Describe roles of funder(s), sponsor(s), and/or institution(s), if any, in developing the protocol
N/A

Section 2: Introduction
Rationale
Item 6. Describe the rationale for the review in the context of what is already known
Introduction section: rationale subheading

Objectives
Item 7. Provide an explicit statement of the question(s) the review will address with reference to participants, interventions, comparators, and outcomes (PICO)
Introduction section: objectives subheading

Section 3: Methods

Eligibility criteria
Item 8. Specify the study characteristics (such as PICO, study design, setting, time frame) and report characteristics (such as years considered, language, publication status) to be used as criteria for eligibility for the review
Methods section: search strategy and eligibility criteria subheadings

Item 9. Describe all intended information sources (such as electronic databases, contact with study authors, trial registers or other grey literature sources) with planned dates of coverage
Methods section: search strategy subheading

Search strategy

Item 10. Present draft of search strategy to be used for at least one electronic database, including planned limits, such that it could be repeated
Appendix

Study records

Item 11a: Data management. Describe the mechanism(s) that will be used to manage records and data throughout the review
Methods section: Selection process, Data-collection process and Data items subheadings

Item 11b: Selection process. State the process that will be used for selecting studies (such as two independent reviewers) through each phase of the review (screening, eligibility, and inclusion in meta-analysis)
Methods section: Data Collection Process and assessing study bias subheadings

Item 11c: Data collection process. Describe planned method of extracting data from reports (such as piloting forms, done independently, in duplicate), any processes for obtaining and confirming data from investigators
Methods section: Data Collection Process

Data items

Item 12. List and define all variables for which data will be sought (such as PICO items, funding sources) and any pre-planned data assumptions and simplifications
Methods: data items subheading

Outcomes and prioritisation

Item 13. List and define all outcomes for which data will be sought, including prioritisation of main and additional outcomes, with rationale
Methods: data items subheading

Risk of bias individual studies

Item 14. Describe anticipated methods for assessing risk of bias of individual studies, including whether this will be done at the outcome or study level, or both; state how this information will be used in data synthesis
Methods: Assessing study bias and Certainty assessment subheadings

Item 15a. Describe criteria under which study data will be quantitatively synthesised
Methods: data synthesis subheading

Item 15b. If data are appropriate for synthesis, describe planned summary measures, methods of handling data, and methods of combining data from studies, including any planned exploration of consistency (such as I^2, Kendall's τ)
Methods: data synthesis subheading

Item 15c. Describe any proposed additional analyses (e.g., sensitivity or subgroup analyses, meta-regression)

N/A

Item 15d. If quantitative synthesis is not appropriate, describe the type of summary planned
Methods: data synthesis subheading

Meta-bias(es)

Item 16. Specify any planned assessment of meta-bias(es) (such as publication bias across studies, selective reporting within studies)
Methods: data synthesis subheading

Confidence in cumulative estimate

Item 17. Describe how the strength of the body of evidence will be assessed (such as GRADE)
Methods: Certainty assessment subheading

For peer review only

PRISMA-P checklist

Section 1: Administrative information

Title

Item 1a: Identification. Identify the report as a protocol of a systematic review:
Title

Item 1b: Update. If the protocol is for an update of a previous systematic review, identify as such
N/A

Registration

Item 2. If registered, provide the name of the registry (such as PROSPERO) and registration number
See below abstract, PROSPERO registration number CRD42023444854

Authors

Item 3a: Contact information. Provide name, institutional affiliation, and email address of all protocol authors; provide physical mailing address of corresponding author
Title page

Item 3b: Contributions. Describe contributions of protocol authors and identify the guarantor of the review
Author contributions section

Amendments

Item 4 If the report represents an amendment of a previously completed or published protocol, identify as such and indicate what changes were made; otherwise state plan for documenting important protocol amendments
N/A

Support

Item 5a: Sources. Indicate sources of financial or other support for the review
Funding statement section

Item 5b: Sponsor. Provide name of the review funder and/or sponsor
Funding statement section

Item 5c: Role of sponsor and/or funder. Describe roles of funder(s), sponsor(s), and/or institution(s), if any, in developing the protocol

N/A

Section 2: Introduction

Rationale

Item 6. Describe the rationale for the review in the context of what is already known

Introduction section: rationale subheading

Objectives

Item 7. Provide an explicit statement of the question(s) the review will address with reference to participants, interventions, comparators, and outcomes (PICO)

Introduction section: objectives subheading

Section 3: Methods

Eligibility criteria

Item 8. Specify the study characteristics (such as PICO, study design, setting, time frame) and report characteristics (such as years considered, language, publication status) to be used as criteria for eligibility for the review

Methods section: search strategy and eligibility criteria subheadings

Item 9. Describe all intended information sources (such as electronic databases, contact with study authors, trial registers or other grey literature sources) with planned dates of coverage

Methods section: search strategy subheading

Search strategy

Item 10. Present draft of search strategy to be used for at least one electronic database, including planned limits, such that it could be repeated

Appendix

Study records

Item 11a: Data management. Describe the mechanism(s) that will be used to manage records and data throughout the review

Methods section: Selection process, Data-collection process and Data items subheadings

Item 11b: Selection process. State the process that will be used for selecting studies (such as two independent reviewers) through each phase of the review (screening, eligibility, and inclusion in meta-analysis)

Methods section: Data Collection Process and assessing study bias subheadings

Item 11c: Data collection process. Describe planned method of extracting data from reports (such as piloting forms, done independently, in duplicate), any processes for obtaining and confirming data from investigators

Methods section: Data Collection Process

Data items

Item 12. List and define all variables for which data will be sought (such as PICO items, funding sources) and any pre-planned data assumptions and simplifications
Methods: data items subheading

Outcomes and prioritisation

Item 13. List and define all outcomes for which data will be sought, including prioritisation of main and additional outcomes, with rationale
Methods: data items subheading

Risk of bias individual studies

Item 14. Describe anticipated methods for assessing risk of bias of individual studies, including whether this will be done at the outcome or study level, or both; state how this information will be used in data synthesis
Methods: Assessing study bias and Certainty assessment subheadings

Item 15a. Describe criteria under which study data will be quantitatively synthesised
Methods: data synthesis subheading

Item 15b. If data are appropriate for synthesis, describe planned summary measures, methods of handling data, and methods of combining data from studies, including any planned exploration of consistency (such as I^2, Kendall's τ)
Methods: data synthesis subheading

Item 15c. Describe any proposed additional analyses (e.g., sensitivity or subgroup analyses, meta-regression)
N/A

Item 15d. If quantitative synthesis is not appropriate, describe the type of summary planned
Methods: data synthesis subheading

Meta-bias(es)

Item 16. Specify any planned assessment of meta-bias(es) (such as publication bias across studies, selective reporting within studies)
Methods: data synthesis subheading

Confidence in cumulative estimate

Item 17. Describe how the strength of the body of evidence will be assessed (such as GRADE)
Methods: Certainty assessment subheading

BMJ Open

Infection and telomere length: a systematic review protocol

Journal:	*BMJ Open*
Manuscript ID	bmjopen-2023-081881.R1
Article Type:	Protocol
Date Submitted by the Author:	15-Mar-2024
Complete List of Authors:	Tunnicliffe, Louis; London School of Hygiene & Tropical Medicine, Faculty of Epidemiology & Population Health<br>Muzambi, Rutendo; London School of Hygiene and Tropical Medicine, Faculty of Epidemiology & Population Health<br>Bartlett, Jonathan; London School of Hygiene & Tropical Medicine, Faculty of Epidemiology & Population Health<br>Howe, Laura; University of Bristol, Social Medicine<br>Abdul Basit, Khalid; London School of Hygiene and Tropical Medicine, Faculty of Epidemiology & Population Health<br>Warren-Gash, Charlotte; London School of Hygiene and Tropical Medicine, Non-communicable disease epidemiology
**Primary Subject Heading**:	Epidemiology
Secondary Subject Heading:	Epidemiology, Infectious diseases, Neurology
Keywords:	Aging, INFECTIOUS DISEASES, NEUROLOGY

SCHOLARONE™
Manuscripts

Infection and telomere length: a systematic review protocol

Authors: Louis Tunnicliffe[1], Rutendo Muzambi[1], Jonathan W. Bartlett[1], Laura D. Howe[2], Khalid Abdul Basit[1], Charlotte Warren-Gash[1]

1. Faculty of Epidemiology & Population Health, London School of Hygiene & Tropical Medicine, Keppel Street, London, UK

2. MRC Integrative Epidemiology Unit, Department of Population Health Sciences, Bristol Medical School, University of Bristol, Oakfield House, Oakfield Grove, Bristol, UK

Corresponding author:
Louis Tunnicliffe Room 256, LSHTM, Keppel Street, London WC1E 7HT Email: louis.tunnicliffe@lshtm.ac.uk Tel: +44(0) 20 7636 8636

Email addresses of protocol authors (in same order as author list above): louis.tunnicliffe@lshtm.ac.uk, rutendo.muzambi@lshtm.ac.uk, jonathan.bartlett1@lshtm.ac.uk, laura.howe@bristol.ac.uk, khalid.abdul-basit@lshtm.ac.uk, charlotte.warren-gash1@lshtm.ac.uk

For peer review only

Abstract: 242/300

Word count: 2329/ 4000

References: 27

Abstract

Introduction- Telomeres are a measure of cellular aging with potential links to diseases such as cardiovascular diseases and cancer. Studies have shown that some infections may be associated with telomere shortening, but whether an association exists across all types and severities of infections and in which populations is unclear. Therefore we aim to collate available evidence to enable comparison and to inform future research in this field.

Methods and analysis- We will search for studies involving telomere length and infection in various databases including MEDLINE (Ovid interface), EMBASE (Ovid interface), Web of Science, Scopus, Global Health, and the Cochrane Library. For grey literature the British Library of electronic theses databases (ETHOS) will be explored. We will not limit by study type, geographic location, infection type or method of outcome measurement. Two researchers will independently carry out study selection, data extraction, and risk of bias assessment using the ROB2 and ROBINS-E tools. Overall quality of the studies will be determined using the Grading of Recommendations, Assessment, Development, and Evaluations criteria. We will also evaluate study heterogeneity with respect to study design, exposure and outcome measurement and if there is sufficient homogeneity, a meta-analysis will be conducted. Otherwise we will provide a narrative synthesis with results grouped by exposure category and study design.

Ethics and dissemination- The present study does not require ethical approval. Results will be disseminated via publishing in a peer-reviewed journal and conference presentations.

PROSPERO registration number CRD42023444854

Strengths and limitations of this study

- This study will be conducted in adherence to the established Preferred Reporting Items for Systematic Reviews and Meta-analyses Protocols (PRISMA-P) statement
- Study selection, data extraction, risk of bias assessment and certainty assessment will be performed by two independent reviewers using well established guidelines and methods
- We will search a range of relevant databases including published and grey literature
- One limitation is that suspected heterogeneity with respect to exposure type and outcome measurement may mean a meta-analysis is not possible

Introduction

Rationale

Telomeres are structures found at the ends of chromosomes which are composed of repetitive DNA sequences and protective proteins. Their primary role is to shield the genomic DNA from being recognized as damaged or broken to prevent processes such as DNA end-joining, DNA recombination, or DNA repair that could lead to chromosome instability (1).

The DNA replication machinery in cells cannot fully copy the DNA at the extreme ends of linear chromosomes, which results in the gradual shortening of chromosome ends with each cell division (1). Eukaryotic cells address this via an enzyme called telomerase which acts to replenish the chromosome ends (2). However in many human cell types, the levels of telomerase (or its activity on telomeres) are limited. This combined with factors such as nuclease action, chemical damage, and DNA replication stress results in the continuous shortening of telomeres throughout a person's lifespan. For this reason, telomere length is used as a measure of biological aging (1).

When telomeres reach a critical length or experience significant damage, a prolonged DNA damage response is triggered. This results in changes to gene expression patterns and leads to cellular senescence (3). The specific outcomes of senescence are thought to vary depending on cell type (1). It has been extensively documented that inflammation plays a significant role in the progression of diseases like cardiovascular disease, chronic kidney disease and Alzheimer’s disease (4). Given that immune cell senescence induces pro-inflammatory processes, telomere attrition in immune cells becomes relevant to the development of these conditions (1). Furthermore there is evidence to suggest that telomere shortening is associated with increased incidence of various diseases including Alzheimer’s disease (5) and cardiovascular disease (6) even after adjusting for age. The idea that shorter telomeres are a potential risk factor for age associated diseases is reinforced by the fact that inherited telomere syndromes, where individuals are genetically pre-disposed to have short telomeres, are characterised by phenotypes of accelerated aging, including a host of age-associated diseases (7).

Telomere length has been shown to be associated with lifestyle and environmental factors (1). For example, early-life connections between stress and telomeres are evident (8). Infections are another factor which could influence telomere length via pathways such as inflammation and oxidative stress (9)(10). However, there is lack of robust evidence relating to the association between infection and telomere length.

While some infections have been studied in relation to telomere length, existing studies differ in the types and severities of infections studied, definitions used and use differing

measures of telomere length, making pooling evidence across studies challenging (9,10)(11-15). Moreover, cross-sectional studies are the most abundant study type in this field; meaning there is a potential for reverse causality. Despite the heterogeneity, some evidence suggests that associations between some persistent viral infections such as Cytomegalovirus and Herpes simplex virus type-1 were associated with reduced telomere length or telomere attrition (9,10,12,13,15). Current gaps in research include establishing whether infections as a whole are risk factors for reduced telomere length and whether pathogen type, severity, and infection site are associated with telomere length. It is plausible that telomere attrition could act as a mechanism through which infections mediate effects on age-related diseases and thereby represent a target for intervention. However, the degree to which any associations are causal remains unclear A systematic review looking at the potential association between infection and telomere length is needed as no prior reviews have been conducted and they are crucial for identifying research gaps and informing the design of future studies.

Objectives

This systematic review aims to comprehensively summarize all existing literature on the association between infections (by type, site, severity) and telomere length or attrition across a broad range of study designs (see eligibility criteria) in adult humans. We aim to establish whether there is an association between infection and telomere length to inform future studies.

Research questions

1. Is there an association between infections and telomere length or attrition?

2. Is infection type, site, severity associated with telomere length or attrition?

3. Is preventing or treating infections associated with telomere length or attrition?

Methods and analysis

The current protocol for our systematic review adheres to the guidelines provided by the Preferred Reporting Items for Systematic Reviews and Meta-analyses Protocols (PRISMA-P) statement and has been registered in the PROSPERO (registration number CRD42023444854) (16,17). Any modifications to the protocol will be documented and updated on PROSPERO.

We intend to follow the PRISMA statement for reporting the systematic review and if applicable employ the Meta-analysis of Observational Studies in Epidemiology statement for the reporting of any potential meta-analysis (18,19).

For peer review only - http://bmjopen.bmj.com/site/about/guidelines.xhtml

Search strategy

We will perform a comprehensive search strategy (search date August 31, 2023) encompassing both published studies and grey literature. Published studies will be sought from six electronic databases, namely MEDLINE (Ovid interface), EMBASE (Ovid interface), Web of Science, Scopus, Global Health, and the Cochrane Library. For grey literature the British Library of electronic theses databases (ETHOS) will be explored. Additionally, the reference lists of included papers will be manually searched to identify any additional relevant studies.

We constructed a preliminary Medline search using three concepts namely 1. infections 2. telomere length and 3. human study type, these search concepts were combined using the Boolean operator 'AND'. Our search involved combining key words with database-specific subject headings and this search can be found in the appendix. This search was created and translated across databases with support from a librarian at the London School of Hygiene and Tropical Medicine. No restrictions were placed on the geographical location, language or date of publication of the studies. We will aim to translate any potentially relevant non-English language studies. The full search strategy can be found in the supplementary information.

Selection process

We will utilize reference management software EndNote (version X8.0.2) for storing our search results. We will conduct de-duplication using the automated feature and subsequently inspect the results to identify and eliminate any duplicate entries manually.

For the selection of studies, two researchers will independently assess all titles and abstracts to determine their agreement with the eligibility criteria described below. Reviewers will discuss and agree which articles should go to full text review. We will then obtain the full texts and the process will be repeated. In the event of discrepancies between the reviewers, we will discuss these and if necessary a third reviewer will be consulted. All reasons for excluding studies will be documented at the full text review stage and our study selection process will be illustrated using the PRISMA flow diagram (18). If multiple papers stem from the same study population then we will include the paper that encompasses the largest sample size and provides the most comprehensive exposure and outcome details.

Eligibility criteria

Studies will be eligible for inclusion in the present study if they meet the criteria below:

Study characteristics

To capture all potentially relevant designs, we will include cross-sectional studies, case control studies, cohort studies, randomised control trials (of vaccination or infection treatment) and Mendelian randomisation studies.

We will include studies of any setting and time-frame.

Population

We will include studies with adults aged ≥18 years from any geographic area and any healthcare / study environment. Animal studies will be excluded.

Exposure

The exposure group will be individuals exposed to infection (i.e. any pathogen, site, severity, type e.g. acute or chronic). Infection diagnosis could be defined through electronic healthcare records (e.g. using ICD-10 or Read coded diagnoses), self-report, antibody measures or other laboratory markers of infection (e.g. PCR). For Mendelian randomisation studies, exposure will be individuals who carry the genetic variants associated with infection and for randomised controlled trials the exposure would be people receiving a vaccine or treatment for infection.

Comparators

The comparator group will vary by study type. For cross-sectional and cohort studies the comparator group will be individuals unexposed to infection. For case-control studies the comparator group is individuals with normal telomere length. For Mendelian randomisation studies, comparators will be individuals who do not carry the genetic variants associated with infection. Finally, for randomised controlled trials the comparator would be people not receiving vaccine or treatment for infection.

Outcome

The outcome will be (i) telomere length (ii) telomere attrition for longitudinal studies. We will be inclusive in how outcome is measured for example; measured by rate of change, continuous measures or binary measures.

We will include studies with any valid method of ascertainment measurement of telomere length. These include PCR (Polymerase Chain Reaction) methods, TRF (Terminal Restriction Fragment) analysis, a variety of FISH (Fluorescence In Situ Hybridization) methods, STELA (Single TElomere Length Analysis), and TeSLA (Telomere Shortest Length Assay) (20). We will not limit by the cell type in which telomere length is measured.

For peer review only

Data Collection Process

Two independent researchers will extract information from the selected papers using a piloted data extraction form. The first reviewer will conduct the data extraction in full whereas the second reviewer will extract data on a 10% random sample of the selected studies. In cases where essential data are missing, we will contact authors to request the necessary information.

Data Items

To create our data extraction form, we will adopt the Population, Exposure, Comparator, Outcomes, and Study Characteristics (PECOS) framework (21). Our data extraction will encompass the following elements:

1. Population: This section will include information about the population under study, such as age (mean, median, or range), gender distribution, and the criteria used for inclusion and exclusion e.g. health conditions, location of residence.

2. Exposure: We will extract details regarding the definition of the exposure, the type of infection involved, whether it relates to hospitalized infection, its acute or chronic nature, and the number of individuals exposed.

3. Comparators: Information related to comparators will encompass their identification, definition, and the count of comparators used in the study.

4. Outcomes: We will collect data on the type of measurement used for telomere length (e.g., binary or continuous) and the number of participants who experienced the specified outcome.

5. Study Characteristics: This section will provide essential details about the study, including the authors' names, the study's title, publication year, study design,

healthcare setting, country where the study was conducted, sample size, and the duration of follow-up.

Furthermore, we will document any collected covariates and effect modifiers and ensure that both unadjusted and adjusted effect estimates and accompanying 95% confidence intervals are included in our data extraction process. We will also include the results of sub-group analyses. e.g. by age and sex.

Assessing Study Bias

Two researchers will independently assess bias following the Cochrane collaboration approach (22) using the ROBINS-E tool (23) for observational studies and the ROB2 (24) tool for randomized controlled trials (RCTs). Both tools will be pilot tested. The ROBINS-E tool will involve evaluating the risk of bias in the following domains: confounding, measurement of the exposure, selection of participants into the study (or into the analysis), post-exposure interventions, missing data, measurement of the outcome, selection of the reported result. The ROB2 tool will involve evaluating bias related to the following domains: the randomisation process, deviations from intended interventions, missing outcome data, measurement of the outcome, selection of the reported result.

Data Synthesis

We will categorize studies based on exposure (stratifying by factors such as infection/pathogen type and site, severity, acute or chronic status), outcome (such as length or attrition, cell type), study type and summarize data in predefined tables. Our primary analyses will focus on the main exposures of any infection, any vaccination and any antimicrobial treatment. We will then conduct secondary analyses of infection type and severity.

A meta-analysis will be considered feasible if there are at least five homogeneous studies in terms of design, exposure (infection/pathogen type, severity), outcome (telomere length measurement technique) as well as the time between exposure and outcome measurement. Pooled effect measures (odds ratios, risk ratios or hazard ratios and corresponding 95% confidence intervals) of the studies will be computed and study results displayed in Forest plots.

Statistical heterogeneity will be assessed using forest plots, χ^2 test, and I^2 statistic and a random effects meta-analysis will be conducted (25-26). Publication bias and small study effects will be assessed with funnel plots if there are ≥10 eligible studies. If a

meta-analysis is unfeasible then a narrative synthesis will be provided with results grouped by exposure.

Certainty assessment

We will use the Grading of Recommendations, Assessment, Development and Evaluation (GRADE) tool to evaluate evidence quality for each outcome (27). Domains considered include risk of bias (determined as described above), inconsistency, indirectness, imprecision, and publication bias. The evidence will be categorized as high, moderate, low, or very low.

Ethics and Dissemination

The present study does not require ethical approval. Results will be submitted for publication in a peer-reviewed journal and may be presented at relevant conferences. The review will highlight research gaps and future directions in this field.

Patient and Public Involvement

Patients and the public were not involved in any way.

Authors contributions:

Louis Tunnicliffe wrote the present paper and constructed the search strategy with the help of a London School of Hygiene & Tropical Medicine librarian. The writing and search strategy was reviewed by Professor Charlotte Warren-Gash, Doctor Rutendo Muzambi, Professor Laura Howe and Professor Jonathan Bartlett. Louis will be conducting the search, data extraction as well as risk of bias and certainty assessments alongside another independent reviewer namely Doctor Khalid Abdul Basit.

Funding statement:

This work was supported by the Wellcome Trust. Grant number: Wellcome Career Development Award 225868/Z/22/Z to Charlotte Warren-Gash.

Competing interests statement:

There are no competing interests to declare.

References

1. Blackburn EH, Epel ES, Lin J. Human telomere biology: A contributory and interactive factor in aging, disease risks, and protection. Science. 2015 Dec 4;350(6265):1193-8. doi: 10.1126/science.aab3389. PMID: 26785477.
2. Blackburn EH, Greider CW, Szostak JW. Telomeres and telomerase: the path from maize, Tetrahymena and yeast to human cancer and aging. Nat Med. 2006 Oct;12(10):1133-8. doi: 10.1038/nm1006-1133. PMID: 17024208.
3. Abdallah P, Luciano P, Runge KW et al. A two-step model for senescence triggered by a single critically short telomere. Nat Cell Biol. 2009 Aug;11(8):988-93. doi: 10.1038/ncb1911. Epub 2009 Jul 13. Erratum in: Nat Cell Biol. 2010 May;12(5):520. PMID: 19597486; PMCID: PMC4025917.
4. Ferrucci L, Fabbri E. Inflammageing: chronic inflammation in ageing, cardiovascular disease, and frailty. Nat Rev Cardiol. 2018 Sep;15(9):505-522. doi: 10.1038/s41569-018-0064-2. PMID: 30065258; PMCID: PMC6146930.
5. Cai Z, Yan LJ, Ratka A. Telomere shortening and Alzheimer's disease. Neuromolecular Med. 2013 Mar;15(1):25-48. doi: 10.1007/s12017-012-8207-9. Epub 2012 Nov 16. PMID: 23161153.
6. Zhan Y, Hägg S. Telomere length and cardiovascular disease risk. Curr Opin Cardiol. 2019 May;34(3):270-274. doi: 10.1097/HCO.0000000000000613. PMID: 30747731.
7. Walne AJ, Dokal I. Advances in the understanding of dyskeratosis congenita. Br J Haematol. 2009 Apr;145(2):164-72. doi: 10.1111/j.1365-2141.2009.07598.x. Epub 2009 Feb 4. PMID: 19208095; PMCID: PMC2882229.
8. Price LH, Kao HT, Burgers DE et al. Telomeres and early-life stress: an overview. Biol Psychiatry. 2013 Jan 1;73(1):15-23. doi: 10.1016/j.biopsych.2012.06.025. Epub 2012 Jul 24. PMID: 22831981; PMCID: PMC3495091.
9. Dowd JB, Bosch JA, Steptoe A et al. Persistent Herpesvirus Infections and Telomere Attrition Over 3 Years in the Whitehall II Cohort. J Infect Dis. 2017 Sep 1;216(5):565-572. doi: 10.1093/infdis/jix255. PMID: 28931225; PMCID: PMC5853283.
10. Noppert GA, Feinstein L, Dowd JB et al. Pathogen burden and leukocyte telomere length in the United States. Immun Ageing. 2020 Nov 19;17(1):36. doi: 10.1186/s12979-020-00206-9. PMID: 33292353; PMCID: PMC7677839.
11. Huang D, Lin S, He J et al. Association between COVID-19 and telomere length: A bidirectional Mendelian randomization study. J Med Virol. 2022 Nov;94(11):5345-5353. doi: 10.1002/jmv.28008. Epub 2022 Jul 29. PMID: 35854470; PMCID: PMC9349767.
12. Pańczyszyn A, Boniewska-Bernacka E, Głąb G. Telomere length in leukocytes and cervical smears of women with high-risk human papillomavirus (HR HPV) infection. Taiwan J Obstet Gynecol. 2020 Jan;59(1):51-55. doi: 10.1016/j.tjog.2019.11.007. PMID: 32039800.
13. Auld E, Lin J, Chang E et al. HIV Infection Is Associated with Shortened Telomere Length in Ugandans with Suspected Tuberculosis. PLoS One. 2016 Sep 21;11(9):e0163153. doi: 10.1371/journal.pone.0163153. PMID: 27655116; PMCID: PMC5031464.
14. Muhsen K, Sinnreich R, Merom D et al. Helicobacter pylori infection, serum pepsinogens as markers of atrophic gastritis, and leukocyte telomere length: a

population-based study. Hum Genomics. 2019 Jul 22;13(1):32. doi: 10.1186/s40246-019-0217-3. PMID: 31331390; PMCID: PMC6647065.

15. van Baarle D, Nanlohy NM, Otto S et al. Progressive telomere shortening of Epstein-Barr virus-specific memory T cells during HIV infection: contributor to exhaustion? J Infect Dis. 2008 Nov 1;198(9):1353-7. doi: 10.1086/592170. PMID: 18816191.
16. Moher D, Shamseer L, Clarke M et al; PRISMA-P Group. Preferred reporting items for systematic review and meta-analysis protocols (PRISMA-P) 2015 statement. Syst Rev. 2015 Jan 1;4(1):1. doi: 10.1186/2046-4053-4-1. PMID: 25554246; PMCID: PMC4320440.
17. Booth A, Clarke M, Dooley G et al. The nuts and bolts of PROSPERO: an international prospective register of systematic reviews. Syst Rev. 2012 Feb 9;1:2. doi: 10.1186/2046-4053-1-2. PMID: 22587842; PMCID: PMC3348673.
18. Moher D, Liberati A, Tetzlaff J et al; PRISMA Group. Preferred reporting items for systematic reviews and meta-analyses: the PRISMA statement. PLoS Med. 2009 Jul 21;6(7):e1000097. doi: 10.1371/journal.pmed.1000097. Epub 2009 Jul 21. PMID: 19621072; PMCID: PMC2707599.
19. Stroup DF, Berlin JA, Morton SC et al. Meta-analysis of observational studies in epidemiology: a proposal for reporting. Meta-analysis Of Observational Studies in Epidemiology (MOOSE) group. JAMA. 2000 Apr 19;283(15):2008-12. doi: 10.1001/jama.283.15.2008. PMID: 10789670.
20. Montpetit AJ, Alhareeri AA, Montpetit M et al. Telomere length: a review of methods for measurement. Nurs Res. 2014 Jul-Aug;63(4):289-99. doi: 10.1097/NNR.0000000000000037. PMID: 24977726; PMCID: PMC4292845.
21. Morgan RL, Whaley P, Thayer KA et al. Identifying the PECO: A framework for formulating good questions to explore the association of environmental and other exposures with health outcomes. Environ Int. 2018 Dec;121(Pt 1):1027-1031. doi: 10.1016/j.envint.2018.07.015. Epub 2018 Aug 27. PMID: 30166065; PMCID: PMC6908441.
22. Higgins JP, Altman DG, Gøtzsche PC et al; Cochrane Bias Methods Group; Cochrane Statistical Methods Group. The Cochrane Collaboration's tool for assessing risk of bias in randomised trials. BMJ. 2011 Oct 18;343:d5928. doi: 10.1136/bmj.d5928. PMID: 22008217; PMCID: PMC3196245.
23. Higgins J, Morgan R, Rooney A et al; ROBINS-E Development Group. Risk Of Bias In Non-randomized Studies - of Exposure (ROBINS-E). Launch version, 20 June 2023. Available from: https://www.riskofbias.info/welcome/robins-e-tool.
24. Sterne JAC, Savović J, Page MJ et al. RoB 2: a revised tool for assessing risk of bias in randomised trials. BMJ 2019; 366: l4898.
25. Riley RD, Higgins JP, Deeks JJ. Interpretation of random effects meta-analyses. BMJ. 2011 Feb 10;342:d549. doi: 10.1136/bmj.d549. PMID: 21310794.
26. Higgins J , Green S . Section 9.5.2. identifying and measuring heterogeneity in Cochrane Handbook for systematic reviews of interventions, 2011. Available: https://handbook-5-1.cochrane.org/chapter_9/9_5_2_identifying_and_measuring_heterogeneity.htm [accessed 30 Oct 2023]
27. Guyatt GH, Oxman AD, Vist GE et al; GRADE Working Group. GRADE: an emerging consensus on rating quality of evidence and strength of recommendations. BMJ.

2008 Apr 26;336(7650):924-6. doi: 10.1136/bmj.39489.470347.AD. PMID: 18436948; PMCID: PMC2335261.

For peer review only

Appendix

Medline search strategy (no limits):

1 (infect* or pathogen or virus* or viral or bacteri* or parasit* or communicable disease*).mp.

2 exp Infections/

3 (telomer* or TTAGGG* or chromosome end* or chromosome cap* or end-replication problem or end-replication malfunction* or end-replication issue* or end-replication impairment* or end-replication failure*).ti,ab.

4 Telomere Shortening/

5 Telomere/

6 ((case* adj5 control*) or (case adj3 comparison*) or control group* or cohort or longitudinal or prospective or retrospective).ti,ab. or "clinical trial".pt. or "clinical trial, phase i".pt. or "clinical trial, phase ii".pt. or clinical trial, phase iii.pt. or clinical trial, phase iv.pt. or controlled clinical trial.pt. or "multicenter study".pt. or "randomi?ed controlled trial".pt. or ((randomi?ed adj7 trial*) or (controlled adj3 trial*) or (clinical adj2 trial*) or ((single or doubl* or tripl* or treb*) and (blind* or mask*))).ti,ab,kw. or ("4 arm" or "four arm").ti,ab,kw. or (cross-sectional or prevalence or transversal).ti,ab,kw. or mendelian randomi?ation.ti,ab. or control patients.mp. or control subjects.mp. or control participants.mp. or patient*.ti,ab. or subjects.ti,ab. or Case-Control Studies/ or Control Groups/ or Matched-Pair Analysis/ or Cohort Studies/ or Longitudinal Studies/ or Follow-Up Studies/ or Prospective Studies/ or Retrospective Studies/ or Double-Blind Method/ or Clinical Trials as Topic/ or Clinical Trials, Phase I as Topic/ or Clinical Trials, Phase II as Topic/ or Clinical Trials, Phase III as Topic/ or Clinical Trials, Phase IV as Topic/ or Controlled Clinical Trials as Topic/ or Randomized Controlled Trials as Topic/ or "Early Termination of Clinical Trials"/ or Multicenter Studies as Topic/ or Cross-Sectional Studies/ or Prevalence/ or Epidemiologic Studies/ or Mendelian Randomization Analysis/ or Observational Study/

7 1 or 2

8 3 or 4 or 5

9 6 and 7 and 8

For peer review only

Database: Ovid MEDLINE(R) ALL <conception to August 31, 2023>
Search Strategy:
1 (infect* or pathogen or virus* or viral or bacteri* or parasit* or communicable disease*).mp.
2 exp Infections/
3 (telomer* or TTAGGG* or chromosome end* or chromosome cap* or end-replication problem or end-replication malfunction* or end-replication issue* or end-replication impairment* or end-replication failure*).ti,ab.
4 Telomere Shortening/
5 Telomere/
6 ((case* adj5 control*) or (case adj3 comparison*) or control group* or cohort or longitudinal or prospective or retrospective).ti,ab. or "clinical trial".pt. or "clinical trial, phase i".pt. or "clinical trial, phase ii".pt. or clinical trial, phase iii.pt. or clinical trial, phase iv.pt. or controlled clinical trial.pt. or "multicenter study".pt. or "randomi?ed controlled trial".pt. or ((randomi?ed adj7 trial*) or (controlled adj3 trial*) or (clinical adj2 trial*) or ((single or doubl* or tripl* or treb*) and (blind* or mask*))).ti,ab,kw. or ("4 arm" or "four arm").ti,ab,kw. or (cross-sectional or prevalence or transversal).ti,ab,kw. or mendelian randomi?ation.ti,ab. or control patients.mp. or control subjects.mp. or control participants.mp. or patient*.ti,ab. or subjects.ti,ab. or Case-Control Studies/ or Control Groups/ or Matched-Pair Analysis/ or Cohort Studies/ or Longitudinal Studies/ or Follow-Up Studies/ or Prospective Studies/ or Retrospective Studies/ or Double-Blind Method/ or Clinical Trials as Topic/ or Clinical Trials, Phase I as Topic/ or Clinical Trials, Phase II as Topic/ or Clinical Trials, Phase III as Topic/ or Clinical Trials, Phase IV as Topic/ or Controlled Clinical Trials as Topic/ or Randomized Controlled Trials as Topic/ or "Early Termination of Clinical Trials"/ or Multicenter Studies as Topic/ or Cross-Sectional Studies/ or Prevalence/ or Epidemiologic Studies/ or Mendelian Randomization Analysis/ or Observational Study/
7 1 or 2
8 3 or 4 or 5
9 6 and 7 and 8

For peer review only

Database: Embase Classic+Embase <conception to 2023 August 31>
Search Strategy:
1 (infect* or pathogen or virus* or viral or bacteri* or parasit* or communicable disease*).mp.
2 exp Infection/
3 (telomer* or TTAGGG* or chromosome end* or chromosome cap* or end-replication problem* or end-replication malfunction* or end-replication issue* or end-replication impairment* or end-replication failure*).ti,ab.
4 telomere shortening/
5 telomere length/
6 telomere/
7 ((case* adj5 control*) or (case adj3 comparison*) or control group* or cohort or longitudinal or prospective or retrospective).ti,ab. or "clinical trial".pt. or "clinical trial, phase i".pt. or "clinical trial, phase ii".pt. or clinical trial, phase iii.pt. or clinical trial, phase iv.pt. or controlled clinical trial.pt. or "multicenter study".pt. or "randomi?ed controlled trial".pt. or ((randomi?ed adj7 trial*) or (controlled adj3 trial*) or (clinical adj2 trial*) or ((single or doubl* or tripl* or treb*) and (blind* or mask*))).ti,ab,kw. or ("4 arm" or "four arm").ti,ab,kw. or (cross-sectional or prevalence or transversal).ti,ab,kw. or mendelian randomi?ation.ti,ab. or control patients.mp. or control subjects.mp. or control

participants.mp. or patient*.ti,ab. or subjects.ti,ab. or case control study/ or control group/ or cohort analysis/ or longitudinal study/ or follow up/ or prospective study/ or retrospective study/ or double blind procedure/ or "clinical trial (topic)"/ or "phase 1 clinical trial (topic)"/ or "phase 2 clinical trial (topic)"/ or "phase 3 clinical trial (topic)"/ or "phase 4 clinical trial (topic)"/ or "controlled clinical trial (topic)"/ or "randomized controlled trial (topic)"/ or "early termination of clinical trial"/ or "multicenter study (topic)"/ or cross-sectional study/ or prevalence/ or epidemiology/ or Mendelian randomization analysis/
8 1 or 2
9 3 or 4 or 5 or 6
10 7 and 8 and 9

Database: Global Health <conception to August 31, 2023>
Search Strategy:
1 (infect* or pathogen or virus* or viral or bacteri* or parasit* or communicable disease*).mp.
2 exp infections/
3 exp infection/
4 (telomer* or TTAGGG* or chromosome end* or chromosome cap* or end-replication problem* or end-replication malfunction* or end-replication issue* or end-replication impairment* or end-replication failure*).ti,ab.
5 telomeres/
6 ((case* adj5 control*) or (case adj3 comparison*) or control group* or cohort or longitudinal or prospective or retrospective).ti,ab. or "clinical trial".pt. or "clinical trial, phase i".pt. or "clinical trial, phase ii".pt. or clinical trial, phase iii.pt. or clinical trial, phase iv.pt. or controlled clinical trial.pt. or "multicenter study".pt. or "randomi?ed controlled trial".pt. or ((randomi?ed adj7 trial*) or (controlled adj3 trial*) or (clinical adj2 trial*) or ((single or doubl* or tripl* or treb*) and (blind* or mask*))).ti,ab. or ("4 arm" or "four arm").ti,ab. or (cross-sectional or prevalence or transversal).ti,ab. or mendelian randomi?ation.ti,ab. or control patients.mp. or control subjects.mp. or control participants.mp. or patient*.ti,ab. or subjects.ti,ab. or case-control studies/ or cohort studies/ or longitudinal studies/ or retrospective studies/ or clinical trials/ or randomized controlled trials/ or cross-sectional studies/ or disease prevalence/ or seroprevalence/ or epidemiological surveys/ or observational studies/
7 1 or 2 or 3
8 4 or 5
9 6 and 7 and 8

For peer review only

Web of Science **conception to August 31, 2023**

1: **TS**=(infect* or pathogen or virus* or viral or bacteri* or parasit* or "communicable disease*")

2: TS=(telomer* or TTAGGG* or "chromosome end*" or "chromosome cap*" or "end-replication problem*" or "end-replication malfunction*" or "end-replication issue*" or "end-replication impairment*" or "end-replication failure*")

3: TS=((case* **NEAR/**5 control*) or (case **NEAR/3** comparison*) or "control group*" or cohort or longitudinal or prospective or retrospective or (randomi?ed **NEAR/7** trial*) or (controlled **NEAR/3** trial*) or (clinical **NEAR/2** trial*) or ((single or doubl* or tripl* or treb*) and (blind* or mask*)) or "4 arm" or "four arm" or "cross-sectional" or prevalence or transversal or "mendelian randomi?ation" or patient* or subjects)

4: **TS**= ("control patients" or "control subjects" or "control participants")

5: #4 OR #3

6: #5 AND #2 AND #1

For peer review only

SCOPUS **conception to August 31, 2023**

Search within	Search
**Article title, Abstract, Keywords**	infect* OR pathogen OR virus* OR viral OR bacteri* OR parasit* OR {communicable disease*}
Article title, Abstract, Keywords	telomer* OR ttaggg* OR {chromosome end*} OR {chromosome cap*} OR {end-replication problem*} OR {end-replication malfunction*} OR

	{end-replication issue*} OR {end-replication impairment*} OR {end-replication failure*}
Article title, Abstract, Keywords	{case* W/5 control* } OR {case W/3 comparison* } OR {control group*} OR cohort OR longitudinal OR prospective OR retrospective OR {randomi?ed W/7 trial* } OR {controlled W/3 trial* } OR {clinical W/2 trial* } OR ((single OR doubl* OR tripl* OR treb*) AND (blind* OR mask*)) OR {4 arm} OR {four arm} OR cross-sectional OR prevalence OR transversal OR {mendelian randomi?ation} OR patient* OR subjects OR participant*

For peer review only

Cochrane: **Conception to August 31, 2023**

1) infect* or pathogen or virus* or viral or bacteri* or parasit* or **(communicable NEXT disease*)**

2) MeSH descriptor: [Infections] explode all trees

3) telomer* or TTAGGG* or (chromosome NEXT end*) or (chromosome NEXT cap*) or (end-replication NEXT problem*) or (end-replication NEXT malfunction*) or (end-replication NEXT issue*) or (end-replication NEXT impairment*) or (end-replication NEXT failure*)

4) MeSH descriptor: [Telomere] explode all trees

5) MeSH descriptor: [Telomere Shortening] explode all trees

6) #1 or #2

7) #3 or #4 or #5

8) #6 and #7

For peer review only

PRISMA-P checklist

Section 1: Administrative information

Title

Item 1a: Identification. Identify the report as a protocol of a systematic review:
Title

Item 1b: Update. If the protocol is for an update of a previous systematic review, identify as such
N/A

Registration

Item 2. If registered, provide the name of the registry (such as PROSPERO) and registration number
See below abstract, PROSPERO registration number CRD42023444854

Authors

Item 3a: Contact information. Provide name, institutional affiliation, and email address of all protocol authors; provide physical mailing address of corresponding author
Title page

Item 3b: Contributions. Describe contributions of protocol authors and identify the guarantor of the review
Author contributions section

Amendments

Item 4 If the report represents an amendment of a previously completed or published protocol, identify as such and indicate what changes were made; otherwise state plan for documenting important protocol amendments
N/A

Support

Item 5a: Sources. Indicate sources of financial or other support for the review
Funding statement section

Item 5b: Sponsor. Provide name of the review funder and/or sponsor
Funding statement section

Item 5c: Role of sponsor and/or funder. Describe roles of funder(s), sponsor(s), and/or institution(s), if any, in developing the protocol

N/A

Section 2: Introduction

Rationale

Item 6. Describe the rationale for the review in the context of what is already known

Introduction section: rationale subheading

Objectives

Item 7. Provide an explicit statement of the question(s) the review will address with reference to participants, interventions, comparators, and outcomes (PICO)

Introduction section: objectives subheading

Section 3: Methods

Eligibility criteria

Item 8. Specify the study characteristics (such as PICO, study design, setting, time frame) and report characteristics (such as years considered, language, publication status) to be used as criteria for eligibility for the review

Methods section: search strategy and eligibility criteria subheadings

Item 9. Describe all intended information sources (such as electronic databases, contact with study authors, trial registers or other grey literature sources) with planned dates of coverage

Methods section: search strategy subheading

Search strategy

Item 10. Present draft of search strategy to be used for at least one electronic database, including planned limits, such that it could be repeated

Appendix

Study records

Item 11a: Data management. Describe the mechanism(s) that will be used to manage records and data throughout the review

Methods section: Selection process, Data-collection process and Data items subheadings

Item 11b: Selection process. State the process that will be used for selecting studies (such as two independent reviewers) through each phase of the review (screening, eligibility, and inclusion in meta-analysis)

Methods section: Data Collection Process and assessing study bias subheadings

Item 11c: Data collection process. Describe planned method of extracting data from reports (such as piloting forms, done independently, in duplicate), any processes for obtaining and confirming data from investigators

Methods section: Data Collection Process

Data items

Item 12. List and define all variables for which data will be sought (such as PICO items, funding sources) and any pre-planned data assumptions and simplifications

Methods: data items subheading

Outcomes and prioritisation

Item 13. List and define all outcomes for which data will be sought, including prioritisation of main and additional outcomes, with rationale

Methods: data items subheading

Risk of bias individual studies

Item 14. Describe anticipated methods for assessing risk of bias of individual studies, including whether this will be done at the outcome or study level, or both; state how this information will be used in data synthesis

Methods: Assessing study bias and Certainty assessment subheadings

Item 15a. Describe criteria under which study data will be quantitatively synthesised

Methods: data synthesis subheading

Item 15b. If data are appropriate for synthesis, describe planned summary measures, methods of handling data, and methods of combining data from studies, including any planned exploration of consistency (such as I^2, Kendall's τ)

Methods: data synthesis subheading

Item 15c. Describe any proposed additional analyses (e.g., sensitivity or subgroup analyses, meta-regression)

N/A

Item 15d. If quantitative synthesis is not appropriate, describe the type of summary planned

Methods: data synthesis subheading

Meta-bias(es)

Item 16. Specify any planned assessment of meta-bias(es) (such as publication bias across studies, selective reporting within studies)

Methods: data synthesis subheading

Confidence in cumulative estimate

Item 17. Describe how the strength of the body of evidence will be assessed (such as GRADE)

Methods: Certainty assessment subheading
